# Criticisms on the Treatment of the Venereal Disease

**Published:** 1802-08-01

**Authors:** T. Vage


### *Criticiſms on the Treatment of the Venereal Diſeaſe,*
### By T. Vage, M. D. F. R S.

[ Continued from Vol. VIII. p. 7 — 13. ]

In conſidering the dyſpeptic ſymptoms of this, or any other diſeaſe, it appears to be generally conceived, that the cauſe of them is the weakneſs of the ſtomach alone. This opinion has probably led to ſome important miſtakes in practice; for this organ is not leſs ſubject to be affected by cauſes, and the condition of parts remote from itſelf, than it is capable of affecting the whole ſyſtem. Thus an indolence of the inteſtines, or a diminution of their action in any part, from the pylorus to the rectum, will produce nauſea and indigeſtion, even when the ſtomach itſelf may be in a good condition. And hence it is, that a cathartic will often remove theſe ſymptoms, by giving an additional irritation to the obſtructed and enervated parts. In general however, here, the ſtomach participates of the mercurial debility, and corroborating aperients become requiſite. In regard to the inertneſs of the inteſtinal action, it may be further noted, that it frequently proceeds from a deficiency of the bile, which a cathartic ſtimulus is likely to prevent; for undoubtedly this ſecretion depends much upon the proper action of the duodenum. But the chief utility of the bile, reſults from its chylific property, which appears to conſiſt, in a great meaſure, of mixing the oily and aqueous parts of the aliment, and aſſimilating them into an uniform liquid. This great importance of the hepatic ſecretion, whenever it appears defective, demands immediate aſſiſtance; and the following pills have been preſcribed, upon ſuch occaſions, with much advantage.

R. Sapon. ven. pulv. rhab. al. ſuccot. aa. ℥j. pulv. arom. ℥ſs. Syr. zinz. q. s. ft. maſs. et form. pil. xij. ex ſingulo, drach.

A few general ideas more, remain yet to be adverted to. And firſt, a free lacteal abſorption, requires a further conſideration, ſtill, than a good condition of the digeſtive organs. It requires to be conſtantly favored by proper evacuations, from the maſs of fluids; for the reſiſtance of a vaſcular plenitude would prevent the influx of the chyle. The cuticular pores, kidnies, and pulmonary exhalations, beſides their emunctorial uſes, are inſervient to this purpoſe. The utmoſt care, therefore, is required to keep them up to their proper quantity. And indeed, this is ſo eaſily accompliſhed by a dry warm air, fit cloathing, exerciſe, and the like, that there is little need for officinal remedies. There is no complaint that affords leſs room for the preſumption of phyſic, than mercurial infirmities.

The

The cold-bath, according to common opinion, as a bracer, may appear ftrictly fuitable to thefe infirmities, but it is extremely apt, not only to caufe others, as, violent head-achs, obftinate rheumatic pains, &c. but alfo to counteract a principal intention of cure, the corroboration of the digeftive vifcera. Even in the common debilitated ftate of the folids, for which it is recommended, it is known to be attended frequently with much differvice; and requires confiderable medical fkill to direct its application. It may therefore, afford fome affiftance, in this refpect, to give a leading idea or two upon the nature and properties of heat: For the effects of cold are nothing elfe but the effects of the abfence or abftraction of this powerful agent, in fome degree or other.

The principal property of fire, which feems not to be commonly confidered, and on which its action chiefly depends, is the repellency of its particles from each other. Another property of fire refulting from the former, is to impart, or rather force its plus from its accumulations, into the contiguous and furrounding bodies which happen to have lefs heat, until all become equal. By this introduction of fire into the interftitial pores of fubftances, they fuffer an expanfion in proportion to the quantity of it, and continue to expand in all their dimenfions, until either they arrive at the fame temperature with the external heat, or until the heat evaporates as faft as it is received, as in boiling water in open veffels. But in oppofition to this expanfion of bodies by fire, the power of attraction between the particles of matter contracts them again, according to the abftraction of their heat.

This increafe, or diminution of fire, in animated bodies, particularly in the human fyftem, produces the following effects. The expanfion of the former, puts the organs of feeling on the ftretch, and according to the degree of it, occafions different degrees of uneafinefs and pain, up to intolerable anguifh. By the contraction which attends any confiderable abftraction of heat, the parts grow dull, torpid, and at laft totally infenfible. In fhort, the communication of additional heat is perhaps the quickeft and moft powerful irritant of the nervous fyftem, and its abftraction the moft effectual fedative; upon which account, it would feem capable of being made a far more extenfive principle of practice than it is at prefent. The effects, however, which follow the abftraction of heat, arife from the conftriction of the parts compreffing the nervous fibres, and their expanfions. But in the human fyftem, befides its fubjection to the reception and diminution of fire, common to other bodies, its nerves appear to be more immediately the conductors of it; for it is certain, that the local application of any any hot or cold fubftance, will produce an additional pofitive general warmth, or a general pofitive diminution of it, much fooner than could be effected through inert, lifelefs matter, by the fame application. Agreeably to this theory, the cold-bath acts, and therefore cannot be fuppofed ferviceable, when the debility of the folids arifes from the indolence of the nervous fyftem. On the other hand, tepid bathing, judicioufly practifed, and particularly the tepid fea-water bath, contributes not a little to the fuccefs of the other parts of the treatment already mentioned.

The fanguific property which Boerhaave afcribes to chalybeates, and their benefit in relaxed habits, was fuppofed a fufficient ground to employ them in thefe cafes; but their utility did not amount to general praife. In fome patients they were attended with manifeft fervice; in others with manifeft detriment. And yet I am inclined to think, that if the mode of their operation was well underftood, it would enable practitioners to ufe them in moft of thefe cafes with advantage. Iron is afcertained by experiment to be capable of conftituting a part of nutritious chyle, in its fartheft elaborations; while other minerals act as extraneous matter in the circulation, and difcompofes the conftitution. Hence workers in this metal are generally remarkably healthy and robuft. May not chalybeates, therefore, in remedial quantities, operate by increafing the natural ftimulus of the blood in the heart and arteries, the additional impetus of which muft promote both the chylific fecretions and the emunctorial ones? And confidered in this nutritious light, as they can be given to invigorate the circulation in any degree, even up to a febrile and inflammatory ftate, may they not be regarded as a fafe and fuccefsful remedy, in all cafes where artificial fever is requifite, which often happens in judicious practice?

The infirmities of the venereal virus itfelf, are more refractory to remedial affiftance than the former; and the inefficacy of the preceding plan of treatment will fhew them to be of that ftamp. They are diftinguifhable alfo from the mercurial debilities, in being more pointed by particular pains and achs after any violent exercife: For in all cafes of venereal ulceration, there is a deftruction of fome of the veffels or fibres of the parts, by which the remaining collateral ones are obliged to perform the offices of the whole; and the unufual diftenfion of them which this requires, cannot fail to occafion difagreeable fenfations and pains. In young people, however, by this very means, the ill effects of venereal corrofion are remedied in time; for where any veffels belonging to a function are deftroyed, the reft become gradually dilated, until, at laft, they give an eafy paffage to the fame quantity of fluids, that the whole at firft conveyed.

*Hydrocele*

